# A novel view of the insulin signaling pathway based on prediction of protein structure by the AI platform AlphaFold

**DOI:** 10.1111/jdi.13988

**Published:** 2023-02-14

**Authors:** Kyosuke Sato, Kenji Sugawara, Wataru Ogawa

**Affiliations:** ^1^ Division of Diabetes and Endocrinology, Department of Internal Medicine Kobe University Graduate School of Medicine Kobe Japan

## Abstract

The predicted structures of major proteins involved in the insulin signaling pathway obtained from the AlphaFold Protein Structure Database.

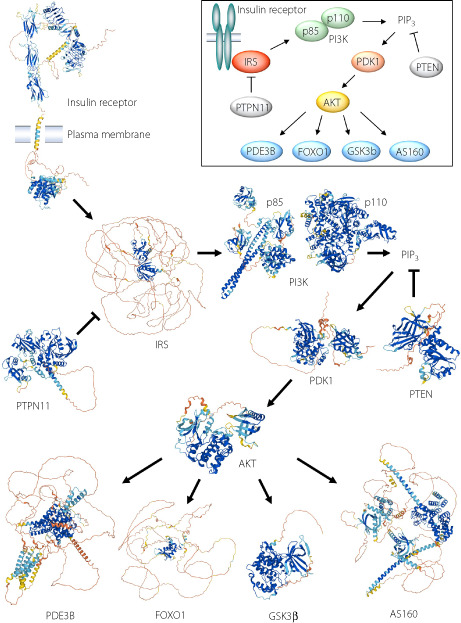

Recent advances in artificial intelligence (AI) are rapidly affecting many aspects of daily life. This new technology has also had a large impact on life science research. In particular, in fields that deal with massive data sets – such as genomics, imaging, and real‐world clinical research – AI is contributing to the diagnosis of or the risk prediction for various diseases as well as to the development of new treatments. One major achievement of AI technology is the prediction of protein folding. Alphafold2, an AI system developed by DeepMind, has revolutionized the field of protein structure by predicting the three‐dimensional architecture of a given protein from only its amino acid sequence with a high level of accuracy^1^, ^2^.

Proteins were long thought to exert their functions in a manner dependent on regions with ‘rigid’ structures. Regions with no defined secondary or tertiary structure, sometimes termed intrinsically disordered regions (IDRs), were thus thought to serve merely as intrinsic connectors of the functional domains^3^. However, it is now clear that, similar to regions with rigid structures, IDRs are subject to various posttranslational modifications, interact with other molecules, and play pivotal roles in protein function^3^.

Signaling initiated by the insulin receptor is one of the most extensively studied intracellular signaling pathways (Figure 1). Binding of insulin to its receptor triggers robust stimulation of the intrinsic tyrosine kinase activity of the receptor and the consequent phosphorylation of insulin receptor substrate (IRS) on tyrosine residues^4^, ^5^. Phosphorylated IRS then binds to other signaling proteins including phosphatidylinositol 3‐kinase (PI3K), which consists of both regulatory and catalytic subunits, as well as the adapter protein Grb2, with the latter interaction leading to activation of the Ras–MAPK (mitogen‐activated protein kinase) pathway^4^, ^5^. The association of IRS with the regulatory subunit of PI3K and Grb2 is mediated by SH2 (Src homology 2) domains of the latter proteins. The binding of IRS to PI3K results in the activation of the lipid kinase and the consequent generation of phosphatidylinositol 3,4,5‐trisphosphate (PIP_3_), which in turn activates the serine–threonine kinase PDK1 (3‐phosphoinositide–dependent kinase 1)^4^, ^5^. PDK1 phosphorylates and thereby activates RAC‐β serine–threonine protein kinase (AKT), which then phosphorylates various target molecules including glycogen synthase kinase 3β (GSK3β), Forkhead box O1 (FOXO1), phosphodiesterase 3B (PDE3B), and AKT substrate of 160 kDa (AS160), which mediate the effects of insulin on glycogen synthesis, gluconeogenesis, lipolysis, and glucose uptake, respectively^4^, ^5^. Protein tyrosine phosphatase nonreceptor type 11 (PTPN11) and phosphatase and tensin homolog deleted from chromosome 10 (PTEN) negatively regulate insulin signaling by mediating the dephosphorylation of tyrosine residues of the insulin receptor or IRS and the 3′ position of PIP_3_, respectively^4^, ^5^. This pathway has been commonly represented as a simplified scheme such as that shown in the inset of Figure 1. However, when the structure of each protein is taken into account – in particular, with discrimination of rigid and nonrigid regions – the pathway can be viewed in a new light.

**Figure 1:**
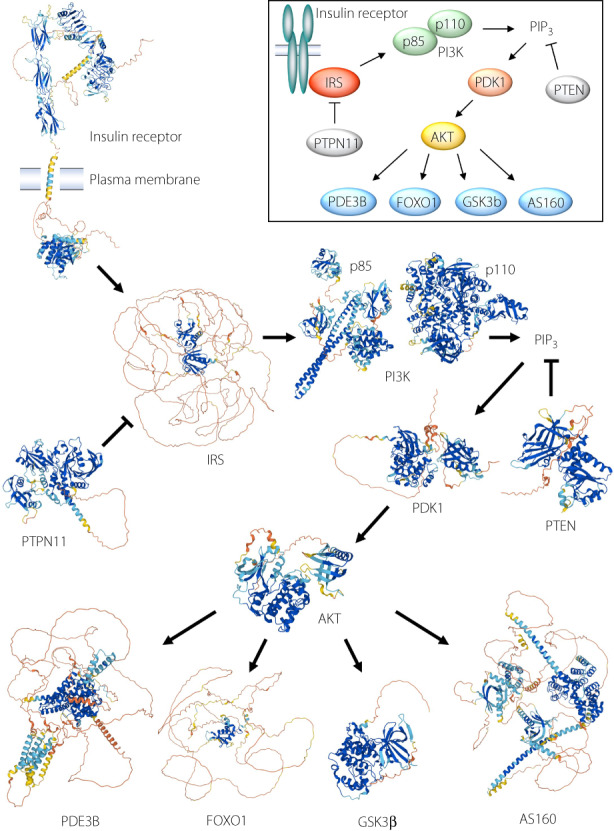
Proteins of the insulin signaling pathway. Three‐dimensional structures and schematic representations of major proteins involved in the insulin signaling pathway are shown in the main panel and inset, respectively. The three‐dimensional structures were obtained from the AlphaFold database (https://alphafold.ebi.ac.uk). Given that the predicted structure of the human insulin receptor in the database does not take into account the orientation of the transmembrane region, the region up to amino acid 952 and that from amino acid 953 are separated and linearly rearranged. In the case of IRS, the structure of IRS1 is shown. More details are provided in Data S1.

Figure 1 shows the predicted structures of major insulin signaling proteins obtained from the AlphaFold Protein Structure Database. Proteins form secondary structures, such as α‐helices and β‐sheets, in a manner dependent on their amino acid sequence, and these regions of secondary structure are then folded to form a three‐dimensional tertiary structure. The AlphaFold database provides predicted secondary and tertiary structures for an enormous number of proteins that are available to the public, allowing even nonexperts to readily obtain predicted structures of proteins of interest. The reliability of predictions in the database is evaluated on the basis of the predicted local distance difference test (pLDDT) score. This score indicates the per‐residue confidence of prediction from 0 to 100, with values of >90 indicating very high confidence and those of <50 very low confidence. The pLDDT score is shown as different colors in the structure, with very high (>90), high (90 to 70), low (70 to 50), and very low (<50) scores being represented in dark blue, light blue, yellow, and red, respectively. In general, regions that form stable secondary and tertiary structures have very high or high pLDDT scores and are thus shown in dark or light blue, and disordered regions have low or very low pLDDT scores and are graphically presented as long filaments of yellow or red^6^.

From such representations, it is readily apparent that kinases and phosphatases – such as PI3K, PDK1, AKT, GSK3β, PTEN, and PTPN11 – consist of rigid conformations throughout almost the entire molecules (Figure 1). This finding is not surprising given that the substrate specificity of the enzymes is likely dependent on unique and distinct structures. Some of these enzymes – including PDK1, GSK3β, and PTPN11 – also possess some nonstructured regions, however, although it is not known whether these regions play specific roles in protein function. PDK1 is activated in response to the generation of PIP_3_ in the plasma membrane and thus serves as a node connecting membrane lipid signaling and a protein kinase cascade in the cytosol. The nonstructured regions of PDK1 may contribute to this role. The α subunit of the insulin receptor is composed of multiple domains with rigid structures linked by nonstructured regions. Insulin binding induces the α subunit of the receptor to undergo a marked conformational change that results in activation of the tyrosine kinase of the β subunit. The relatively long linking regions may facilitate this conformational change of the α subunit. Whereas the tyrosine kinase domain of the β subunit forms a rigid structure, a long nonstructured region is present between the transmembrane region and the tyrosine kinase domain. This nonstructured region, known as the juxtamembrane region, becomes phosphorylated in response to insulin binding to the receptor and serves as a binding site for IRS. The autophosphorylation sites of the β subunit are distributed widely from the juxtamembrane region to the far COOH‐terminal region. The IDR may therefore increase the mobility of the tyrosine kinase domain, allowing autophosphorylation at such multiple sites within the protein.

In marked contrast to the kinases and phosphatases of the insulin signaling pathway, IRS consists mostly of IDRs, the exceptions being the small PH (pleckstrin homology) and PTB (phosphotyrosine binding) domains at the NH_2_‐terminus (Figure 1). These two domains mediate interactions with other proteins, including the insulin receptor. However, given that most regions of IRS are unstructured, it seems likely that this characteristic is important for the function of IRS.

One property of IDRs that has attracted wide attention is the facilitation of liquid–liquid phase separation (LLPS). LLPS gives rise to compartmentalization in cells as a result of the aggregation of proteins and other biopolymers and the consequent formation of droplet‐like structures. Such structures increase the efficiency of biochemical reactions, the sequestration of specific factors, and the organization of associated intracellular structures^7^. The compartmentalized spaces generated by LLPS are known as biomolecular condensates, or simply as liquid droplets. Given their lack of a membrane, liquid droplets readily associate and dissociate, contributing to the dynamism of biochemical reactions. Those formed by proteins involved in signal transduction have been termed signalosomes and can be regarded as membrane‐less organelles. Weak and reversible interactions mediated by IDRs play a key role in the formation of lipid droplets and the dynamics of LLPS^7^.

A recent study showed that the self‐aggregation of IRS *via* its COOH‐terminal IDR triggers LLPS and the formation of signalosomes containing PI3K and Grb2^8^. Furthermore, a mutation of IRS (G972R in IRS1) that is located in this IDR and is associated with the risk of type 2 diabetes impairs the self‐aggregation of IRS and signalosome formation. In addition to PI3K and Grb2, IRS binds to many other signaling proteins, including NCK1, CRK, and Fyn. These IRS binding proteins also contain IDRs that connect protein‐interacting domains such as SH2 and SH3 domains and which likely contribute to aggregation and condensation of multiple signaling proteins (Figure 2a). The structural features of these proteins may therefore contribute to IRS‐mediated LLPS and signalosome formation. Given these considerations, the old‐fashioned scheme for illustrating the interaction of IRS with its binding partners (Figure 2b) should be revised to take into account signalosome formation (Figure 2c).

**Figure 2:**
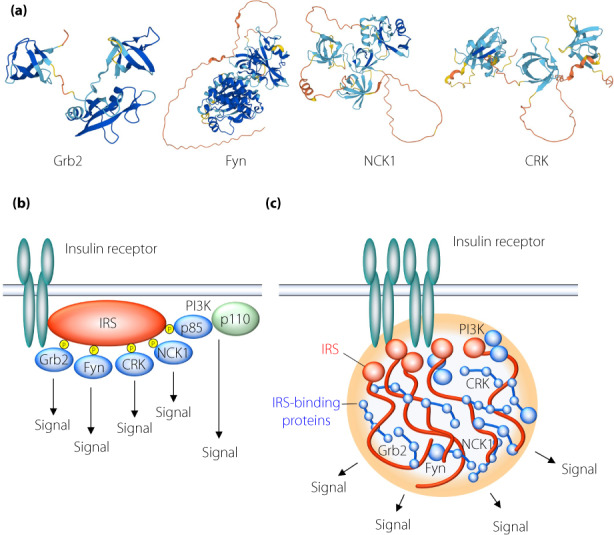
IRS binding proteins. (a) Three‐dimensional structures of IRS binding proteins. (b) Conventional schematic representation of IRS‐mediated signal transduction. (c) Conceptual diagram of signal transduction mediated by IRS‐dependent signalosome formation.

The transcription factor FOXO1 is also notable for its high content of nonstructured regions (Figure 1). Whereas a small deoxyribonucleic acid (DNA) binding domain (the Forkhead domain) possesses a tertiary structure, most other regions of the protein appear to have no defined structure. Most transcriptional factors and cofactors possess long IDRs, with recent studies having revealed that the aggregation of nuclear proteins mediated by such IDRs results in LLPS that promotes transcriptional regulation by increasing the density and facilitating the proper placement of these proteins^9^. AKT‐mediated phosphorylation of FOXO1 results in its export from the nucleus to the cytosol. Given that the phosphorylation sites reside in IDRs and that phosphorylation of IDRs is thought to influence LLPS^10^, such LLPS might play a role in the AKT‐mediated regulation of FOXO1.

Whereas they are shorter than those of IRS or FOXO1, both PDE3B and AS160, which are also substrates of AKT, possess relatively long IDRs (Figure 1). Evidence suggests that 14–3‐3, a small protein that binds to various phosphorylated proteins, regulates the function of both PDE3B^11^ and AS160^12^ through direct binding. Whereas 14–3‐3 itself possesses a rigid structure, the binding motifs for 14–3‐3 in other proteins, including PDE3B and AS160, exist mostly within IDRs. 14–3‐3 is thought to serve as a linker for different IDRs by undergoing self‐dimerization, and has thus been implicated in the induction of LLPS^13^. It will be of interest to examine whether LLPS contributes to insulin signaling mediated by PDE3B and AS160.

The predicted structures that are now readily accessible in the AlphaFold database provide novel insight into the functions and interactions of proteins of interest. It should be noted, however, that Alphafold2 does not always accurately discriminate between structured and nonstructured regions. The possibility that regions predicted to be unstructured may actually have a specific rigid structure therefore cannot be completely excluded. Despite this limitation, Alphafold2 has shed light on aspects of the protein landscape that had previously remained in the dark. Looking at the structures of multiple proteins in one view, as in this article, provides a notion of functional differences and interactions among such proteins that participate in a given pathway and may therefore lead to new scientific discoveries. AI technology in this field will undoubtedly evolve rapidly and may soon be able to predict not only protein structure but also the interactions among all proteins in a given signaling pathway at a detailed spatiotemporal level, including whether such proteins undergo LLPS. Such technological progress will not eliminate the role of the scientist, but it will alter the direction of his or her work, as is the case for all endeavors affected by innovative AI technology.

## DISCLOSURE

The author declares no conflict of interest.

## Supporting information


**Data S1** Information for proteins of the insulin signaling pathway.
