# Supplementary material for: A novel view of the insulin signaling pathway based on prediction of protein structure by the AI platform AlphaFold
Source: J Diabetes Investig. 2023 Feb 14;14(5):635–9. doi: 10.1111/jdi.13988 (PMC10119909; doi:10.1111/jdi.13988)
Supplement: Supplementary file 1 — Data S1 Information for proteins of the insulin signaling pathway. [file JDI-14-635-s001.docx]

**Supplementary Information**

**Information for proteins of the insulin signaling pathway**

INSR, insulin receptor (Uniprot ID: P06213); IRS, insulin receptor substrate 1 (P35568); PI3K, phosphatidylinositol 3-kinase (p85, regulatory subunit α (P27986); p110, catalytic subunit α (P42336)); PDK1, 3-phosphoinositide dependent kinase 1 (O15530); PTPN11, protein tyrosine phosphatase nonreceptor type 11 (Q06124); PTEN, phosphatase and tensin homolog deleted from chromosome 10 (P60484); AKT, RAC-β serine-threonine protein kinase 2 (P31751); PDE3B, phosphodiesterase 3B (Q13370); FOXO1, Forkhead box protein O1 (Q12778); GSK3β, glycogen synthase kinase 3β (P49841); AS160, AKT substrate of 160 kDa (O60343); Grb2, growth factor receptor–bound protein 2 (P62993); Fyn, protein tyrosine kinase Fyn (P06241); NCK1, noncatalytic region of tyrosine kinase adaptor protein 1 (P16333); CRK, adaptor protein CRKII (P46108).
